# Beneficial Plant Microorganisms Affect the Endophytic Bacterial Communities of Durum Wheat Roots as Detected by Different Molecular Approaches

**DOI:** 10.3389/fmicb.2019.02500

**Published:** 2019-10-31

**Authors:** Monica Agnolucci, Michela Palla, Caterina Cristani, Noemi Cavallo, Manuela Giovannetti, Maria De Angelis, Marco Gobbetti, Fabio Minervini

**Affiliations:** ^1^Department of Agriculture, Food and Environment, University of Pisa, Pisa, Italy; ^2^Department of Soil, Plant and Food Science, University of Bari Aldo Moro, Bari, Italy; ^3^Faculty of Science and Technology, Free University of Bozen-Bolzano, Bolzano, Italy

**Keywords:** wheat, Arbuscular mycorrhizal symbionts, *Funneliformis mosseae*, *Lactobacillus plantarum*, root endophytic bacterial biota

## Abstract

This study aimed at characterising the endophytic bacterial communities living in durum wheat roots, as affected by wheat cultivar and inoculation of the Arbuscular mycorrhizal fungus *Funneliformis mosseae* IMA1 and the wheat root endophytic bacterium *Lactobacillus plantarum* B.MD.R.A2. These microorganisms were inoculated, alone or in combination, in durum wheat (cultivars Odisseo and Saragolla). Non-inoculated plants of each cultivar represented the controls. Forty-three days after sowing, roots were deprived of the epiphytic microbiota and subjected to DNA extraction. The DNA was used as template in PCR-DGGE analysis of the 16S rRNA gene (variable region V3–V5) and 16S (region V1–V3) metagenetics. Odisseo and Saragolla root endophytic bacterial biotas differed for number of OTUs and composition. In detail, *Pseudomonas* was higher in Odisseo than in Saragolla. The inoculation of *F. mosseae* and *L. plantarum* increased the abundance of *Pseudomonas*, some Actinobacteria (e.g., *Streptomyces*, *Microbacterium*, two genera including several plant growth promoting (PGP) strains) and Bacteroidetes in both cultivars. However, the endophytic bacterial biota of Saragolla roots inoculated just with lactobacilli did not differ from that of the control. The inoculation of Saragolla with *F. mosseae*, alone or in combination with lactobacilli, led to higher abundance of *Rhodococcus*, belonging to Actinobacteria and encompassing PGP strains. First, this work showed that *F. mosseae* and *L. plantarum* shape the endophytic bacterial biota of durum wheat roots. Abundance of some OTUs was affected by the microbial inoculation, depending on the cultivar. This result represents a starting point for exploitation of beneficial endophytes of wheat roots.

## Introduction

Soil microorganisms have been increasingly recognised as key providers of multiple ecosystem services and essential elements for the completion of biogeochemical cycles, at the basis of long-term soil productivity and health, playing important roles also in human nutrition and welfare (Philippot et al., 2013; Avio et al., 2018). The majority of such beneficial microbes live in the rhizosphere, a complex and dynamic ecosystem harbouring high numbers of diverse plant growth promoting (PGP) microbial communities, including Arbuscular mycorrhizal fungi (AMF, *Glomeromycota*) and their associated bacteria, which positively interact with plant roots (Philippot et al., 2013; Turrini et al., 2018).

Arbuscular mycorrhizal fungi establish mutualistic symbioses with the roots of about 80% of terrestrial plants, including the most important food crops, such as wheat, maize, rice, soybean, bean, clover, alfalfa, fruit trees, vegetables, herbs and medicinal plants and economically important species, such as sunflower, sugarcane, cotton, tobacco, coffee, tea, rubber, and cassava. In exchange for plant carbon (up to 20%), AMF transfer soil mineral nutrients, such as phosphorus (P), nitrogen (N), sulfur (S), potassium (K), calcium (Ca), copper (Cu), and zinc (Zn), from the soil to the host plant, by means of a fine network of extraradical hyphae which grow from mycorrhizal roots into the surrounding soil (Smith and Read, 2008). Beyond mineral nutrition, AMF provide other benefits, improving plant tolerance to biotic and abiotic stresses, such as pathogenic fungi, salinity and drought, thus reducing the need of chemical fertilisers and pesticides (Gianinazzi et al., 2010).

The diverse bacterial communities living in the mycorrhizosphere, i.e., associated with mycorrhizal roots, spores, sporocarps and extraradical hyphae, were identified by culture-independent methods and affiliated with *Cellvibrio*, *Chondromyces*, *Flexibacter*, *Lysobacter*, *Pseudomonas* (Roesti et al., 2005), Proteobacteria and Actinobacteria (Long et al., 2008), Micrococcales (e.g., *Arthrobacter*), Bacillales (e.g., *Bacillus*, *Paenibacillus*), Rhizobiales (e.g., *Rhizobium*, *Ensifer* formerly known as *Sinorhizobium*), Pseudomonadales (e.g., *Pseudomonas*), Burkholderiales (e.g., *Herbaspirillum*, *Massilia*), *Streptococcus* and *Ensifer* (Agnolucci et al., 2015). Culture-dependent approaches allowed the physiological characterisation of AMF-associated bacterial communities, revealing their multifunctional activities as PGP bacteria, i.e., promotion of AMF activity, control of soil pathogens, nitrogen fixation, nutrient supply and production of growth factors (Xavier and Germida, 2003; Li et al., 2007; Bharadwaj et al., 2008; Battini et al., 2016, 2017). Such studies suggested the occurrence of synergistic interactions with AMF, possibly leading to further positive effects on plant growth, nutrition and health, and to the utilisation of the best performing strains and consortia as biocontrol agents, biofertilizers, and bioenhancers (Rouphael et al., 2015; Turrini et al., 2018).

Some rhizospheric and mycorrhizospheric bacteria are able to colonise plant roots, thus becoming endophytes, either obligate, facultative or passive (Hardoim et al., 2008). Bacteria that derive from seeds and cannot survive in soils are obligate endophytes, while facultative (*alias* opportunistic) endophytes may colonise or not plant tissues, depending on different variables and environmental conditions (Compant et al., 2010). Passive endophytes are bacteria that are able to colonise plant roots just through wounds and cracks (Christina et al., 2013). The prevalent phyla of root endophytic bacteria are represented by Proteobacteria (50% in relative abundance), Actinobacteria (10%), Firmicutes (10%), and Bacteroidetes (10%), but other phyla, such as Acidobacteria, Armatimonadetes, Chloroflexi, Cyanobacteria, Nitrospirae, Planctomycetes, Verrucomicrobia (Sessitsch et al., 2012; Edwards et al., 2015; Ferrando and Fernández Scavino, 2015; Marques et al., 2015; Liu et al., 2017) may be encountered. Endophytic bacterial communities confer multiple beneficial effects to the host plant, promoting plant growth, producing phytohormones, such as auxins, gibberellins, cytokinins, and controlling plant pathogens, via antibiosis, induced systemic resistance or siderophore production (Compant et al., 2010; Hardoim et al., 2015; Santoyo et al., 2016).

Lactic acid bacteria (LAB), such as *Lactococcus lactis* ssp. *lactis*, *Lactobacillus brevis*, *Lactobacillus paracasei* ssp. *paracasei*, *Lactobacillus plantarum*, have been isolated from the rhizosphere of different plant species, including mulberry, mango, vine, banana, starfruit, longan (Chen et al., 2005) and are well represented within the endophytic bacterial communities of strawberry and sugar beet roots (Jacobs et al., 1985; de Melo Pereira et al., 2012). LAB have been proposed as PGP bacteria, given their ability to promote plant growth by increasing nutrient availability from organic material and to protect plant from biotic and abiotic stresses (Lamont et al., 2017). Recent studies revealed that LAB may be found in the endophytic microbiota of durum wheat, with *L. plantarum*, a key-species for several ecosystems including sourdough, associated with roots, leaves, and spikes during wheat life cycle (Minervini et al., 2015). Durum wheat (*Triticum turgidum* ssp. *durum*), one of the most important food crops worldwide, has been studied for its susceptibility to mycorrhizal colonisation, with 108 accessions characterised by the identification of genetic markers associated to such a trait (De Vita et al., 2018). Nothing is known about the multitrophic and possibly synergistic interactions among AMF and their associated bacteria, LAB and durum wheat. Here, as the first step of a comprehensive research plan, we characterised the endophytic bacterial communities living in durum wheat roots, as affected by wheat cultivar and presence/absence of AMF and LAB. To this aim, we utilised the AMF isolate *Funneliformis mosseae* IMA1 and the LAB strain *L. plantarum* B.MD.R.A2. In order to avoid shortfalls linked to the constraints of cultivation conditions, as well as to the presence of bacteria in viable but non-cultivable state, we utilised two culture-independent approaches, namely PCR-DGGE analysis of the 16S ribosomal RNA (rRNA) gene and 16S metagenetics through Illumina MiSeq platform. Our data provide knowledge of different factors shaping the composition of endophytic bacterial communities of durum wheat roots, opening the way to the exploitation of beneficial plant endophytes in sustainable management strategies.

## Materials and Methods

### Arbuscular mycorrhizal Fungal Inoculation

Mycorrhizal treatments were set up using inoculation consisting of mycorrhizal roots and soil containing spores and extraradical mycelium of the AMF species *F. mosseae* (T. H. Nicolson and Gerd.) C. Walker and A. Schüssler (formerly known as *Glomus mosseae*), maintained in the collection of Microbiology Labs (International Microbial Archives, IMA) of the Department of Agriculture, Food and Environment, University of Pisa, Italy (isolate number IMA1). The holotype is deposited in the herbarium of the Department of Biology, University of Pisa, Herbarium Horti Botanici Pisani as PI-HM-Z4. The inoculation was obtained from *Medicago sativa* L. pot cultures in a mixture of sandy loam soil and calcinated clay (OILDRI, Chicago, IL, United States) (1:1 v/v) which was steam-sterilised (121°C for 30 min on two consecutive days) to kill naturally occurring AMF. Chemical and physical characteristics of the soil used were as follows: pH (H_2_O), 8.0; clay, 15.3%; silt, 30.2%; sand, 54.5%; organic matter, 2.2% (Walkley-Black); total N, 1.1 g kg^–1^ (Kjeldahl); extractable P, 17.6 mg kg^–1^ (Olsen). Sterile seeds of *M. sativa* L. were sown, and plants grown for 4 months, then shoots were excised and roots chopped into fragments. Then the substrate was air dried at room temperature and utilised as crude inoculation. The activity of such inoculum was verified by a mycorrhizal inoculum potential (MIP) bioassay (Njeru et al., 2014). MIP values, determined on *Cichorium intybus* host plants, were on average 40–50%, showing that the AMF inoculation was active.

### Bacterial Inoculation

*Lactobacillus plantarum* B.MD.R.A2 is deposited in the Culture Collection of the Departament of Soil, Plant and Food Sciences, University of Bari, as DISSPA-75. This bacterial strain, an endophytic component of roots of durum wheat, isolated from Saragolla roots (Minervini et al., 2015), was cultured on Sourdough Bacteria broth (SDB) (Kline and Sugihara, 1971) at 28°C for 48 h. Bacterial cells were centrifuged (3100 × *g*, 10 min) washed twice in sterile physiological saline solution (0.9% NaCl) and finally suspended in physiological solution, as described in Battini et al. (2017). Bacterial density of the suspension was assessed using a Thoma cell chamber and adjusted to inoculate the soil substrate at sowing with 10^6^ CFU g^–1^.

### Plant and Experimental Conditions

The experiment consisted of four treatment groups: control, AMF inoculum (M), bacterial inoculum (B), AMF and bacterial inoculum (BM). The microcosm experiment was carried out using 50 mL Falcon tubes. The growth substrate was prepared mixing (1:1 v/v) the *F. mosseae* crude inoculation with sterile calcinated clay. Control and bacterial treatments received a mock inoculation produced by sterilising the appropriate amount of mycorrhizal inoculation. All tubes received 2 mL of a filtrate, obtained by sieving the mycorrhizal inoculation through a 50-μm pore diameter sieve and a Whatman paper no. 1 (Whatman International Ltd., Maidstone, Kent, United Kingdom), to ensure common microbiota for all treatments. *L. plantarum* B.MD.R.A2 was inoculated at sowing of durum wheat (*Triticum turgidum* ssp. *durum*) seeds and 15 days after germination using 1 mL of the bacterial suspension per tube, while the same amount of physiological saline solution was provided to treatments not inoculated with lactobacilli.

Before microbial inoculation, durum wheat seeds of the cultivars Odisseo (O) and Saragolla (S) were surface sterilised twice in 1.5% (v/v) sodium hypochlorite solution for 10 min and thoroughly rinsed with sterile MilliQ water. Three seeds were sown in each tube, which was put in sun-transparent bags and maintained in a growth chamber at 27°C under a 16/8 h light/dark daily cycle until harvest. Plantlets were thinned to one per tube at emergence. Six replicates were performed per each treatment. The plantlets were watered as needed. Forty-three days after sowing, plants were harvested, roots removed from soil and washed with tap water. Three replicate plants were used for assessing mycorrhizal colonisation and the other three for extraction of total DNA. In order to measure the level of mycorrhizal colonisation, roots were cleared in 10% (w/v) KOH in a 80°C water bath for 15 min and stained with Trypan blue in lactic acid (0.05%) after 10 min in 2% aqueous HCl. The percentage of AMF colonisation was calculated using a dissecting microscope at ×25 or ×40 magnification and the gridline intersect method (Giovannetti and Mosse, 1980).

### DNA Extraction From Roots

Before DNA extraction, 10 g of plant roots were washed and surface sterilised as described in Minervini et al. (2015). Genomic DNA was extracted using FastDNA Pro Soil-Direct kit (MP Biomedicals, Santa Ana, CA, United States) as described by Minervini et al. (2010). Absorbance values at 260 nm and 280 nm were measured by means of the NanoDrop ND- 1000 (Thermo Fisher Scientific Inc.), in order to assess the concentration and the purity (absorbance at 260 nm/absorbance at 280 nm) of DNA extracts. Three replicate extractions were performed from the three different root samples. The extracted DNA was pooled and used for culture-independent analyses, PCR-DGGE and Illumina MiSeq.

### PCR-DGGE Analysis

For the analysis of endophytic bacterial communities, the amplification of the variable region V3–V5 of 16S ribosomal DNA (rDNA) was carried out using the primers 341F (CCTA CGGGAGGCAGCAG) and 907R (CCGTCAATTCCTTTRAG TTT) (Yu and Morrison, 2004). At its 5′ end, the primer 341F had an additional 40-nucleotide GC-rich tail (5′-CGCC CGCCGCGCCCCGCGCCCGTCCCGCCGCCCCCGCCCG-3′). Amplification reaction was prepared as previously reported (Agnolucci et al., 2015).

For the DGGE analysis, amplicons were separated in 8% (w/v) polyacrylamide gels with a 30–70% urea-formamide gradient, using the DCode^TM^ Universal Mutation Detection System (Bio-Rad, Milan, Italy). A composite mix of bacterial 16S rRNA gene fragments from *L. plantarum* IMA B23, *L. brevis* DSMZ 20054, *Pseudomonas fluorescens* 19/5, *Streptomyces* sp. IMA AC3, *Ensifer meliloti* IMA TSA26, *Fictibacillus barbaricus* IMA TSA50, *Streptomyces* sp. IMA W64 were added as reference DGGE marker (M). Gels were run and visualised as described in Agnolucci et al. (2013). The main bands of DGGE profiles were excised from the gels for sequencing at the Eurofins Genomics MWG Operon (Ebersberg, Germany) as reported in Palla et al. (2018).

### Analysis of Bacterial Diversity Through 16S Metagenetics

PCR was performed at RTL Genomics (Lubbock, TX, United States), using primers 28F/519R, targeting a region spanning from V1 to V3 of Bacteria (Handl et al., 2011). The Illumina 2 × 300 bp paired-end MiSeq platform at RTL Genomics was used to sequence the PCR products. Sequenced reads were processed as follows: (i) merged through the PEAR Illumina paired-end read merger (Zhang et al., 2014); (ii) chimaeras were removed through the UCHIME software (Edgar et al., 2011); (iii) sequences were aligned using the USEARCH global alignment algorithm (Edgar, 2010); and (iv) Operational Taxonomic Units (OTUs) were selected through the UPARSE OTU selection algorithm (Edgar, 2013). OTUs were identified using a NCBI database containing high quality sequences.

### Statistical Analyses

DGGE profiles were digitally processed with BioNumerics software version 7.6 (Applied Maths, St-Martens-Latem, Belgium) as reported in Turrini et al. (2017). A position tolerance and optimisation of 1%, respectively, were used. DGGE patterns were analysed using Unweighted Pair Group Method Using Arithmetic Average (UPGMA) as clustering method, and non-metric multidimensional scaling analysis (NMDS) as reported in Turrini et al. (2017). DGGE banding data were used to estimate biodiversity indices (Richness, Shannon-Weaver, Simpson and Evenness), as reported in Turrini et al. (2017). DGGE band sequences were analysed using BLAST on the NCBI web^1^. The related sequences were collected and aligned using MUSCLE (Edgar, 2004a, b), and phylogenetic trees were constructed using the Neighbour-Joining method based on Kimura’s 2-parameter model (Kimura, 1980) in Mega 6 software^2^ with 1000 bootstrap replicates. Metagenetic data were subjected to permutation analysis using PermutMatrix and Principal Components Analysis (PCA) using Statistica 7.0 for Windows.

### Nucleotide Sequence Accession Numbers

The sequences obtained by PCR-DGGE bands were submitted to the European Nucleotide Archive under the accession numbers from LR536406 to LR536425. The 16S rRNA gene sequences obtained from 16S metagenetic analysis are available in the Sequence Read Archive of NCBI (accession number PRJNA543396).

## Results

### Mycorrhizal Colonisation and Communities of Endophytes Analysed by PCR-DGGE

*Funneliformis mosseae* successfully established mutualistic symbiosis with durum wheat roots, with percentages of colonised root length ranging from 8% to 27%, with no differences between the two cultivars Odisseo and Saragolla. No colonisation was observed in non-inoculated plants.

**FIGURE 1:**
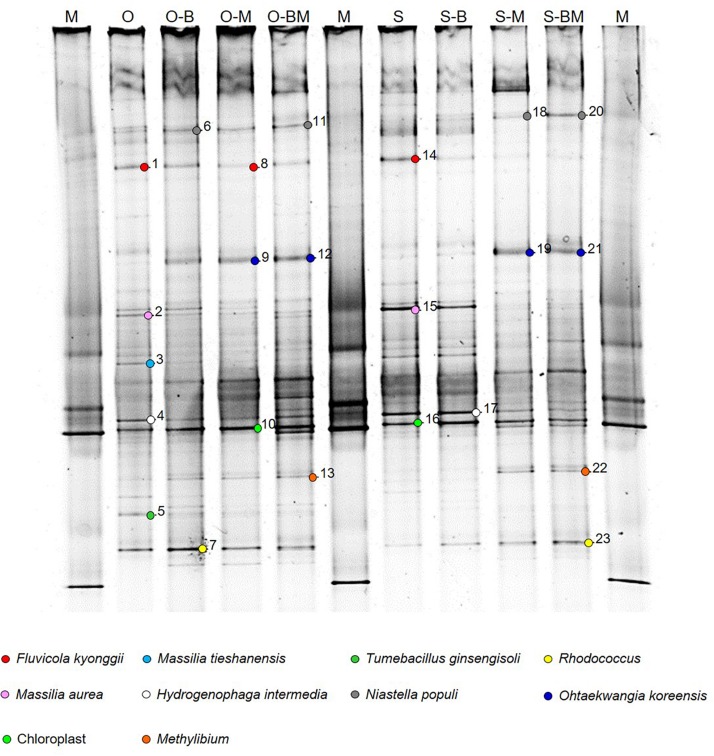
DGGE analysis of endophytic bacterial communities associated with durum wheat (cultivars Odisseo, O, and Saragolla, S) roots inoculated with either *Lactobacillus plantarum* (O-B, S-B), or *Funneliformis mosseae* (O-M, S-M), or *L. plantarum* and *F. mossae* (O-BM, S-BM). Controls (O, S) refer to roots of durum wheat not inoculated. Sequenced fragments are marked with progressive numbers. The three lanes labelled as “M” refer to a laboratory-prepared reference marker (detailed in the paragraph “PCR-DGGE Analysis”).

**FIGURE 2:**
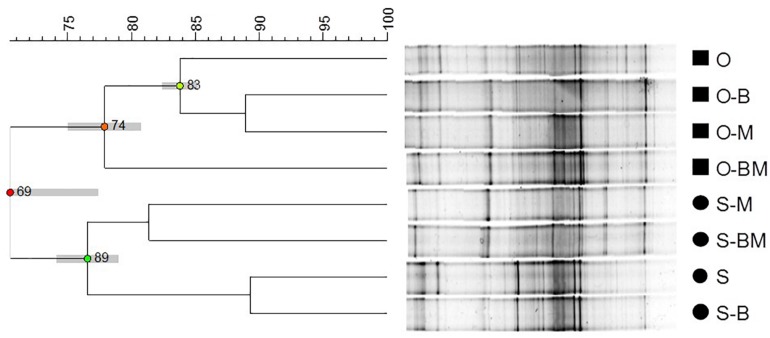
Dendrogram obtained from Unweighted Pair Group Method Using Arithmetic Average (UPGMA) analysis, using Dice’s coefficient, of DGGE profiles of endophytic bacterial communities associated with durum wheat (cultivars Odisseo, O, and Saragolla, S) roots inoculated with either *Lactobacillus plantarum* (O-B, S-B), or *Funneliformis mosseae* (O-M, S-M), or *L. plantarum* and *F. mossae* (O-BM, S-BM). Controls (O, S) refer to roots of durum wheat not inoculated. Cophenetic correlation is shown at each branch by numbers and coloured dots, ranging between green-yellow-orange-red, according to decreasing values. Standard deviation is shown at each node by a grey bar.

DNA fragments of approximately 600 bp, corresponding to the V3–V5 region of the 16S rRNA gene, were successfully amplified from all samples. DGGE analyses of PCR products showed complex profiles characterised by intense and clearly defined fragments (Figure 1). The endophytic community composition of durum wheat root samples was studied by cluster analysis of DGGE profiles (Figure 2). In particular, the relevant dendrogram separated the endophytic community associated with the two cultivars (Odisseo and Saragolla) in two clusters, showing a similarity of 70%. Within the cluster corresponding to the cultivar Odisseo, inoculated samples were differentiated from non-inoculated control (O). In particular, LAB- (O-B) and AMF-inoculated (O-M) samples clustered together at a similarity level of 89%, being separated from control by 84%, while samples co-inoculated with LAB and AMF (O-BM) branched separately with a lower similarity level of 78%.

The cluster corresponding to the cultivar Saragolla was further split into two sub-clusters (77% similarity). The first one contained samples inoculated with AMF (S-M) and co-inoculated with AMF and LAB (S-BM) showing a similarity of 81%, while the second one grouped control (S) and samples inoculated with LAB (S-B), at a similarity of 89%.

**FIGURE 3:**
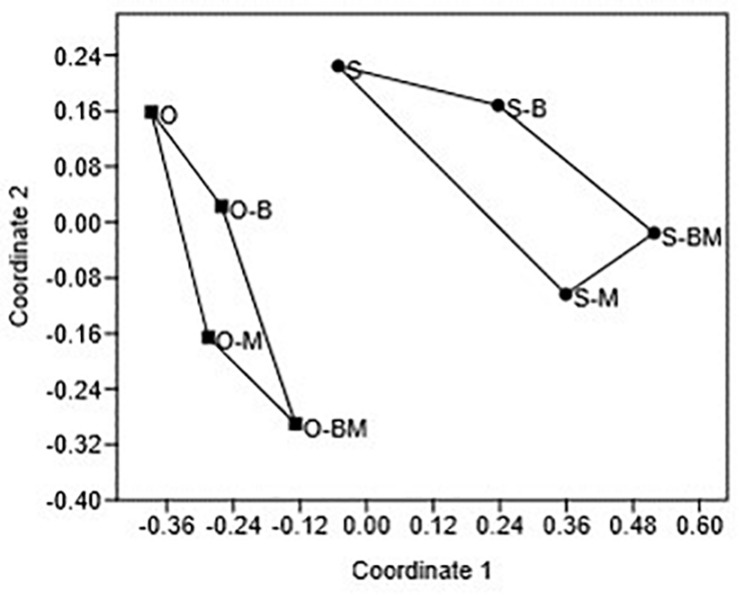
Non-metric multidimensional scaling (NMDS) plot of DGGE profile analysis using Euclidean coefficient. Each point on the plot represents endophytic bacterial communities associated with durum wheat (cultivars Odisseo, O, and Saragolla, S) roots inoculated with either *Lactobacillus plantarum* (O-B, S-B), or *Funneliformis mosseae* (O-M, S-M), or *L. plantarum* and *F. mossae* (O-BM, S-BM). Controls (O, S) refer to roots of durum wheat not inoculated. The stress value is 0.14, the ANOSIM values (R) indicates significant differences between the two cultivars (*R* = 0.7448, *p* < 0.05).

The differences observed between the endophytic community composition associated with the two durum wheat cultivars were also confirmed by NMDS analysis of the DGGE profiles (Figure 3). ANOSIM revealed that such differences were statistically significant (*R* = 0.7448, *p* < 0.05).

Biodiversity indices of PCR-DGGE profiles did not differ among the treatment groups (data not shown).

**FIGURE 4:**
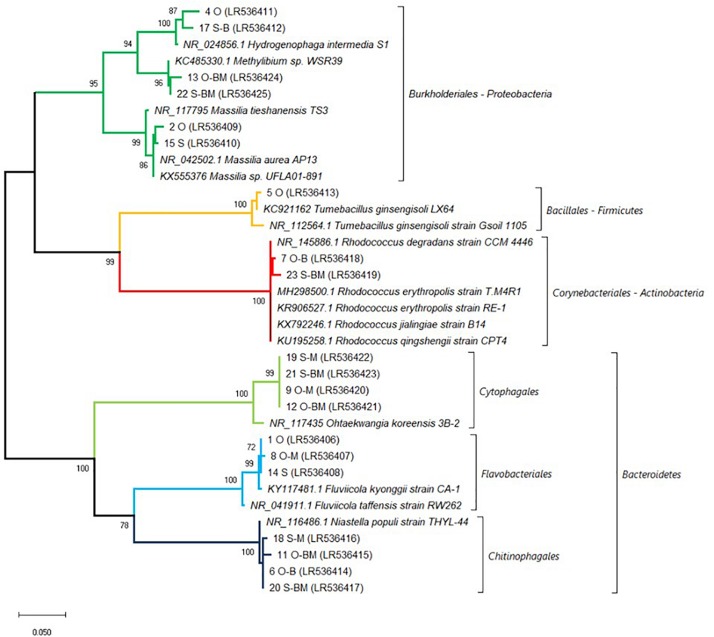
Affiliation of the sequences retrieved from DGGE gel fragments (marked in Figure 1) with the existing sequences of V3–V5 region of 16S rRNA gene. Phylogenetic analysis was inferred by using the Maximum Likelihood method based on the kimura 2-parameter model. Bootstrap (1000 replicates) values below 70 are not shown. Evolutionary analyses were conducted in MEGA X (Kumar et al., 2018). The sequences from the database are indicated by their accession numbers. The DNA sequences retrieved in this work are indicated by their corresponding band number and their accession number. Non-inoculated Saragolla (S), non-inoculated Odisseo (O), inoculated with *Lactobacillus plantarum* (B), inoculated with *Funneliformis mosseae* (M) and co-inoculated (BM).

In order to identify the major bacterial species characterising the endophytic communities analysed, the bands of interest (Figure 1) were excised from DGGE profiles, sequenced and affiliated to species by using BLAST and phylogenetic trees analyses. Sequences belonged to four different phyla: Proteobacteria (*Massilia tieshanensis, Massilia aurea, Methylibium*, and *Hydrogenophaga intermedia*), Firmicutes (*Tumebacillus ginsengisoli*), Actinobacteria (*Rhodococcus*), and Bacteroidetes (*Fluvicola kyonggii*, *Niastella populi* and *Ohtaekwangia koreensis*) (Figures 1, 4).

In particular, the main endophytic species characterising control Odisseo roots were represented by *N. populi*, *F. kyonggii*, *M. aurea*, *M. tieshanensis*, *H. intermedia*, *T. ginsengisoli*, and *Rhodococcus*. The inoculated samples were characterised by higher and lower intensity bands corresponding to *O. koreensis* and *Massilia* and *T. ginsengisoli*, respectively, compared with the non-inoculated control.

The main endophytic species retrieved in Saragolla roots differed from those found in Odisseo, mainly for the absence of *T. ginsengisoli*. Moreover, in Saragolla control *N. populi* and *Rhodococcus* were less abundant. In the inoculated samples, with the exception of S-B, *N. populi*, *O. koreensis*, *Methylibium*, and *Rhodococcus* were well represented, while the bands corresponding to *F. kyonggii*, *H. intermedia*, and *Massilia* species were less intense (Figure 1).

### Communities of Endophytes Analysed by 16S Metagenetics

**FIGURE 5:**
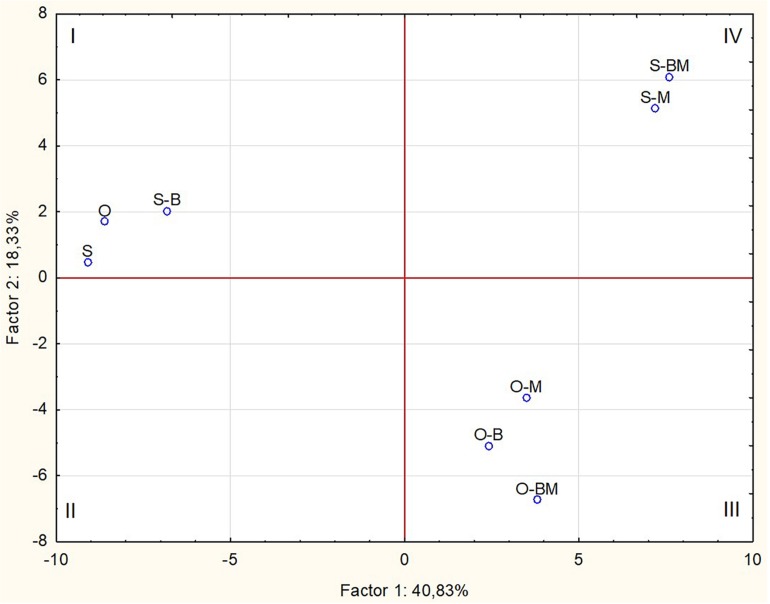
Score plot of first and second principal components after Principal Component Analysis based on bacterial OTUs found through 16S metagenetic analysis of DNA extracted from roots of durum wheat (cultivars Odisseo, O, and Saragolla, S) inoculated with either *Lactobacillus plantarum* (O-B, S-B), or *Funneliformis mosseae* (O-M, S-M), or *L. plantarum* and *F. mossae* (O-BM, S-BM). Controls (O, S) refer to roots of durum wheat not inoculated.

**FIGURE 6:**
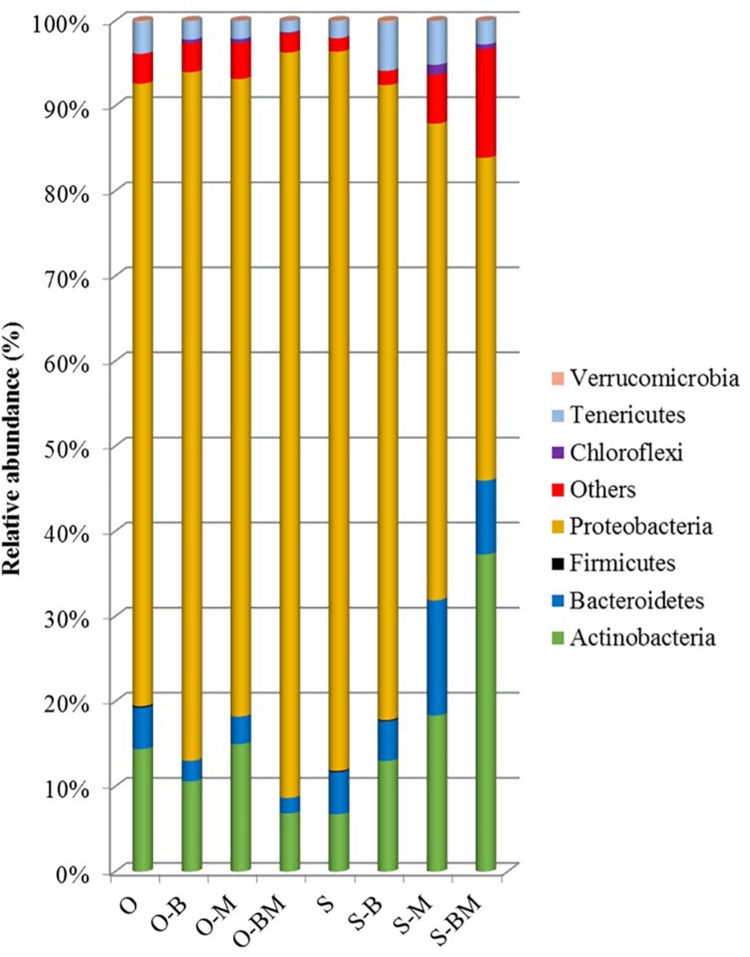
Bacterial phyla distribution (%) found in roots of durum wheat (cultivars Odisseo, O, and Saragolla, S) inoculated with either *Lactobacillus plantarum* (O-B, S-B), or *Funneliformis mosseae* (O-M, S-M), or *L. plantarum* and *F. mossae* (O-BM, S-BM). Controls (O, S) refer to plants of durum wheat not inoculated. “Others” refer to OTUs with a relative abundance ≤0.1% in all the samples.

**FIGURE 7:**
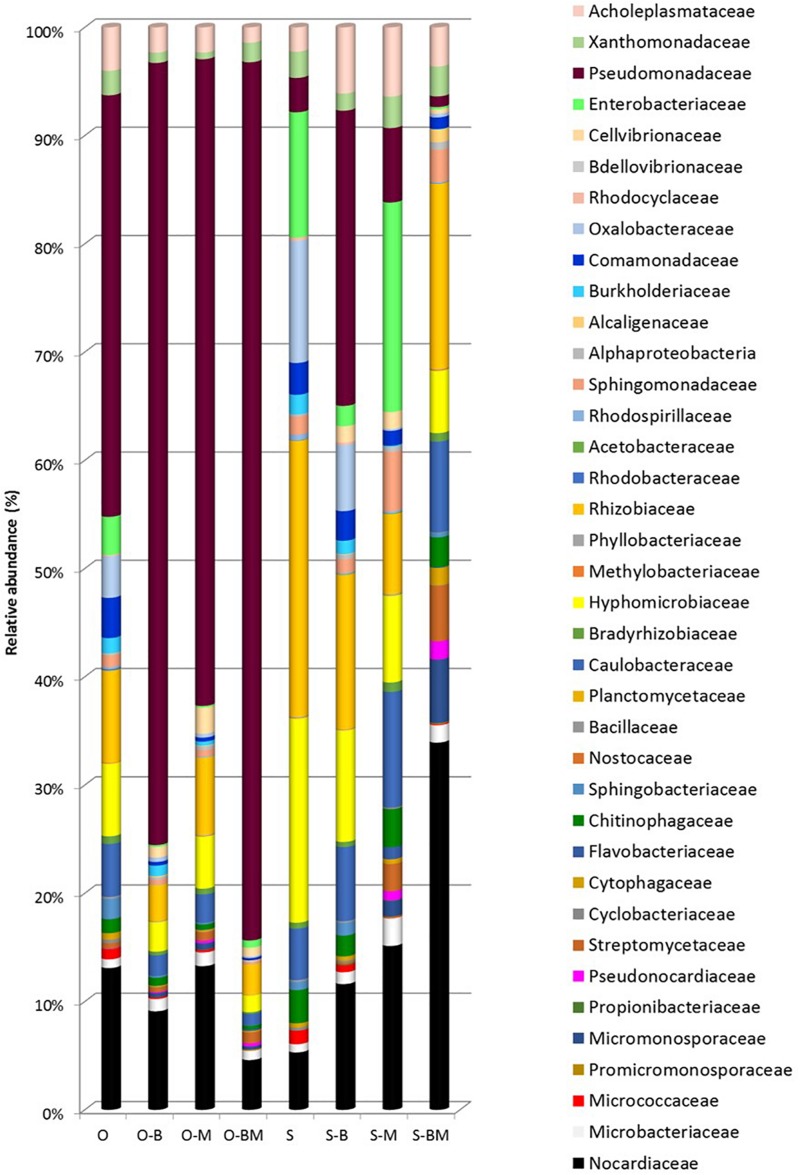
Bacterial families distribution (%) found in roots of durum wheat (cultivars Odisseo, O, and Saragolla, S) inoculated with either *Lactobacillus plantarum* (O-B, S-B), or *Funneliformis mosseae* (O-M, S-M), or *L. plantarum* and *F. mossae* (O-BM, S-BM). Controls (O, S) refer to plants of durum wheat not inoculated. “Others” refer to OTUs with a relative abundance ≤0.1% in all the samples.

**FIGURE 8:**
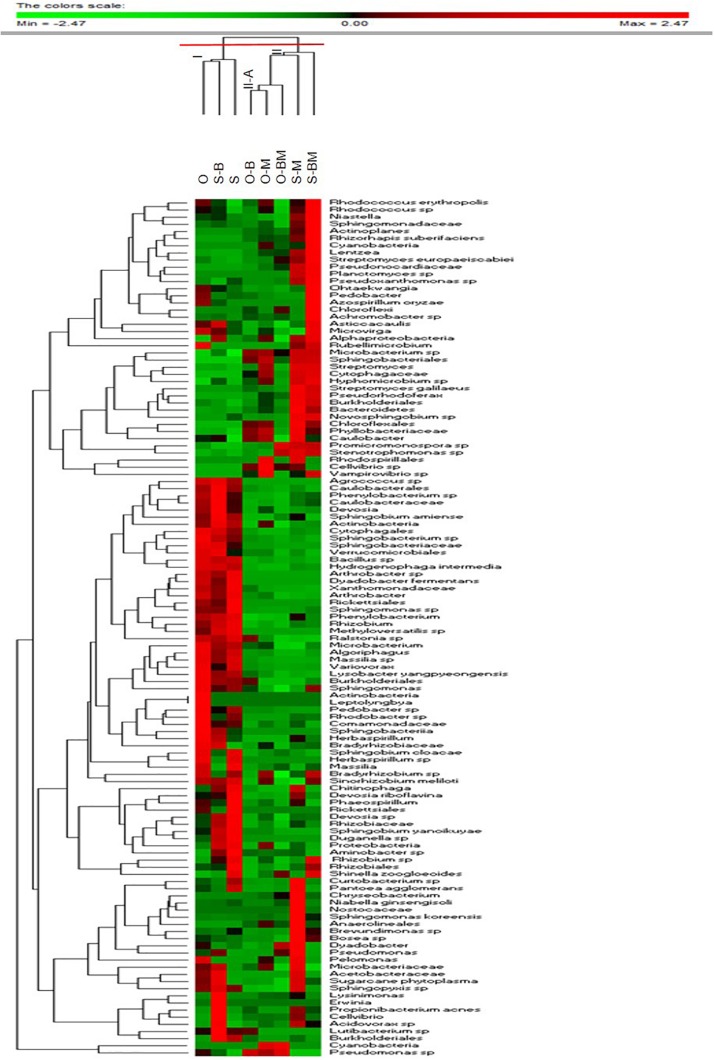
Permutation analysis summarising the relative abundance of the bacterial OTU found in roots of durum wheat (cultivars Odisseo, O, and Saragolla, S) inoculated with either *Lactobacillus plantarum* (O-B, S-B), or *Funneliformis mosseae* (O-M, S-M), or *L. plantarum* and *F. mossae* (O-BM, S-BM). Controls (O, S) refer to plants of durum wheat not inoculated. Euclidean distance and Mc-Quitty’s criterion were used for clustering. Colours correspond to normalised mean data levels from low (green) to high (red).

Total DNA from the endophytic fraction of wheat roots was used as template to amplify the V1–V3 region of 16S rRNA gene. Amplicons were subjected to high throughput sequencing on the Illumina MiSeq platform. A total of 264,504 quality-trimmed sequences of 16S rRNA gene amplicons were obtained, with an average of 33,063 sequences/sample, and an average length of 509 bp (data not shown). The Good’s Estimated Sample Coverage was higher than 99.9% for all the samples (Supplementary Table S1). Control samples of Odisseo and Saragolla harboured a different number of OTUs. The AMF and LAB inoculum increased the OTU number in Saragolla roots. A different behaviour was observed for Odisseo roots. Values of Chao1 richness and Abundance-based Coverage Estimate were in agreement with the number of OTUs. The lowest value of Shannon index (1.37) was found for endophytic bacterial biota in the roots of O-BM. Within the Odisseo samples, 56 OTU, out of a total of 102, were shared by all the treatments, whereas 11 OTU were unique to control Odisseo (Supplementary Figure S1A), in agreement with the results of PCR-DGGE. Fifty-five OTU, out of a total of 104, were shared by all the Saragolla samples (Supplementary Figure S1B). Three and one OTUs were unique to Saragolla roots inoculated with AMF and with LAB, respectively. PCA, based on all the sequences detected, showed that AMF inoculation strongly differentiated the endophytic bacterial communities of both wheat cultivars from the relevant controls, confirming PCR-DGGE cluster analysis. In particular, bacterial endophytes of non-inoculated (control) Odisseo were clearly differentiated from those of the same cultivar inoculated with AMF, LAB or both (Figure 5). Bacterial biota of non-inoculated (control) Saragolla roots fell in the same quadrant (first) as non-inoculated Odisseo. Roots of Saragolla inoculated with AMF or AMF and LAB showed a distinct bacterial endophytic community with respect to non-inoculated roots and to all the other root samples. Proteobacteria were dominant in all samples (Figure 6). Actinobacteria, Bacteroidetes, Tenericutes, Verrucomicrobia, Cloroflexi, and Firmicutes were the other commonly detected phyla. However, compared to controls (non-inoculated roots), Saragolla roots inoculated with AMF and LAB showed the lowest and highest relative abundance of Proteobacteria and Actinobacteria, respectively. Remarkable variations were found when distribution of bacterial families, genera and species was considered (Figures 7, 8 and Supplementary Table S2). Permutation analysis of the relative abundance of OTU grouped samples mainly in two clusters, confirming the strong differentiation of root endophytic bacterial communities between plants inoculated with AMF and the controls, in accordance with PCR-DGGE clustering (Figures 2, 8). The highest similarity was found for inoculated roots of Odisseo (Cluster II-A). Specific bacterial profiles were detected. In detail, the relative abundance of Pseudomonadaceae (mainly *Pseudomonas* genus) strongly differed between Odisseo (35.8%) and Saragolla (ca. 2.8%) roots (Figures 7, 8 and Supplementary Table S2). The inoculation of the AMF *F. mosseae* or the LAB *L. plantarum* increased the relative abundance of Pseudomonadaceae in both cultivars. The co-inoculation of both *F. mosseae* and *L. plantarum* resulted in a further increase of Pseudomonadaceae (ca. 77.2%) only in Odisseo roots.

Rhizobiaceae harboured higher relative abundance in Saragolla than in Odisseo roots (ca. 22.6 vs. 7.8%, respectively). The main OTU component of Rhizobiaceae was the genus *Rhizobium*. The inoculation of *L. plantarum* and/or *F. mosseae* resulted in a decrease of the relative abundance of Rhizobiaceae. Hyphomicrobiaceae (mainly *Devosia*), Oxalobacteraceae (*Duganella* and *Massilia* genera) and Enterobacteriaceae (*Pantoea*) were the other dominant Proteobacteria in Saragolla roots. These families decreased in roots inoculated with *L. plantarum* and/or *F. mosseae*. The only exception was *Pantoea agglomerans*, which was found at the highest level in Saragolla roots inoculated with *F. mosseae* (Figure 8 and Supplementary Table S2). Sub-dominant families were variously found among root samples. Within them, Caulobacteraceae (e.g., *Asticcacaulis*, *Brevundimonas* sp.), found in the same relative amount in the non-inoculated roots (ca. 4.5%), decreased in inoculated Odisseo roots and increased in *L. plantarum* and/or *F. mosseae* inoculated Saragolla roots. Saragolla roots harboured higher Sphingomonadaceae than Odisseo roots. The relative abundance of some species belonging to Sphingomonadaceae (e.g., *Sphingomonas koreensis*) was the highest in roots of Saragolla inoculated with *F. mosseae* (Figures 7, 8 and Supplementary Table S2). Cellvibrionaceae (*Cellvibrio* genus) was detected only in *L. plantarum* and/or *F. mosseae* inoculated Odisseo and Saragolla roots. On the contrary, Burkholderiaceae (*Ralstonia* sp.) disappeared in Odisseo and Saragolla roots co-inoculated with *L. plantarum* and *F. mosseae*. Comamonadaceae, including *Hydrogenophaga intermedia*, was found at the highest level in non-inoculated Odisseo and Saragolla roots.

Within Actinobacteria, the relative abundance of Nocardiaceae (mainly *Rhodococcus* genus) strongly differed between Odisseo (12%) and Saragolla (ca. 4.7%) roots. The inoculation of roots with *L. plantarum* and/or *F. mosseae* led to a decrease of *Rhodococcus* species (*R. erythropolis* and *Rhodococcus* sp.) in Odisseo. Opposite effect was found in the inoculated Saragolla roots. Other Actinobacteria families were variously detected at sub-dominant level among samples. Within them, Microbacteriaceae (*Microbacterium*), Micromonosporaceae (*Actinoplanes*), and Streptomycetaceae (*Streptomyces europaeiscabiei*) increased in inoculated Odisseo and Saragolla roots. Opposite trend was found for Micrococcaceae (*Arthrobacter*).

Cyclobacteriaceae and Cytophagaceae, belonging to Bacteroidetes, decreased in inoculated Odisseo and Saragolla roots. The only exception was for Cytophagaceae (namely *Ohtaekwangia*) which was found at the highest level in Saragolla roots inoculated with *L. plantarum* and *F. mosseae*, as revealed also by PCR-DGGE sequencing (Figures 1, 4). Flavobacteriaceae (*Chryseobacterium*) was detected only in inoculated samples. Chitinophagaceae and Sphingobacteriaceae decreased in inoculated Odisseo roots. In Saragolla roots similar trend was observed for Sphingobacteriaceae. On the contrary, Chitinophagaceae, with the genus *Niastella*, increased in *F. mosseae* and, especially, in Saragolla roots co-inoculated with AMF and LAB, confirming PCR-DGGE findings (Figure 1).

Bacillaceae belonging to Firmicutes, decreased in all the inoculated roots. Acoleplasmataceae, with the *Sugarcane phytoplasma*, was the only identified family of the Tenericutes. *S. phytoplasma* showed a different behaviour between Odisseo and Saragolla roots. Indeed, it decreased in Odisseo inoculated roots while increased in inoculated Saragolla roots.

## Discussion

This work represents the first study revealing that two variables, cultivar and AMF and/or *L. plantarum* inoculation, were able to shape the composition of endophytic bacterial communities of durum wheat roots. The two wheat cultivars Odisseo and Saragolla differed for OTU numbers and bacterial endophytes composition, as assessed by PCR-DGGE and Illumina MiSeq. Remarkably, mycorrhizal inoculation with *F. mosseae* strongly affected the endophytic bacterial communities in both wheat cultivars.

The levels of mycorrhizal colonisation, ranging from 8 to 27%, compare well with previous data obtained with 108 durum wheat accessions inoculated with *F. mosseae*, whose mean values ranged from 6.6 to 22% (De Vita et al., 2018), confirming wheat susceptibility to AMF symbiosis.

The two culture-independent methods, PCR-DGGE and 16S metagenetic analysis, detected strong qualitative and quantitative differences in the composition of root endophytic bacterial communities between the two *T. durum* cultivars Odisseo and Saragolla. In particular, Odisseo roots were characterised by a higher abundance of Pseudomonadaceae (mainly *Pseudomonas* genus) than Saragolla, which showed a high diversity, without dominant taxa. Such results complement and support previous data on the composition of rhizosphere bacteria of wheat, varying with genotype (Donn et al., 2015). Besides genotype, several other factors influence the taxonomic structure of endophytic bacterial community: host species, organ, developmental stage, growing season, geographical location, soil type, nutrient status, cultivation techniques (Liu et al., 2017). Plants play a pivotal role in the recruitment of endophytic bacterial community. In detail, rhizosphere is the first ecosystem that selects bacteria from bulk soil. Further selection occurs at rhizoplane level, where bacteria possessing adhesion ability are potential candidates that enter endosphere. The recruitment process ends thanks to the plant immune system, which actively blocks some specific bacteria (see Santoyo et al., 2016 for review). In this study the root endophytic bacterial biota was mainly composed by Proteobacteria (73 and 84% in Odisseo and Saragolla), Actinobacteria and Bacteroidetes, while Firmicutes, Chloroflexi, Cyanobacteria, Tenericutes, and Verrumicrobia were subdominant, consistently with previous findings (Sessitsch et al., 2012; Edwards et al., 2015; Ferrando and Fernández Scavino, 2015; Marques et al., 2015; Liu et al., 2017).

The inoculation of *F. mosseae* and *L. plantarum* of wheat plants affected the root endophytic community composition of Odisseo and Saragolla, increasing the abundance of some genera and species of Actinobacteria and Bacteroidetes, as suggested by the Venn’s diagrams (Supplementary Figure S1). A different behaviour was shown by Saragolla roots inoculated with LAB. In detail, the endophyte composition did not differ from that of the control, as revealed by PCR-DGGE clustering and confirmed by PCA based on the results of 16S metagenetics (Figures 1, 2, 5). This finding is very interesting, when considering the origin of the endophytic strain *L. plantarum* B.MD.R.A2, isolated from Saragolla roots (Minervini et al., 2015), whose inoculation did not greatly affect the bacterial endophyte communities.

In Odisseo roots, inoculation of AMF or LAB resulted in a higher abundance of *Pseudomonas* in the bacterial endophytic community, which was further increased when AMF and LAB had been co-inoculated. It is important to note that many strains of different *Pseudomonas* species have long been known as PGP bacteria, playing also a key role as biocontrol agents (Schlatter et al., 2017). Inoculation of *Pseudomonas fragi* CS11RH1 (MTCC 8984) on wheat plants enhanced phosphate solubilisation, indolacetic acid production, rate of seed germination and plant biomass (Selvakumar et al., 2009), while the strain EPS 1 increased the accumulation of Zn in the grain and root dry weight (Kamran et al., 2017). Moreover, *Pseudomonas aeruginosa* protected wheat plants against oxidative stress induced by Zn, through the improvement of nutrients bioavailability, lowering of Zn uptake and elicitation of plant antioxidant responses (Islam et al., 2014).

In Saragolla roots, inoculation with *F. mosseae* alone or in combination with *L. plantarum* led to an increase of Actinobacteria, in particular *Rhodococcus* species (*R. erythropolis* and *Rhodococcus* sp.), while the abundance of other genera, such as *Streptomyces* and *Microbacterium* spp., was enhanced in both cultivars. Actinobacteria, frequently isolated from the soil, are considered key soil beneficial bacterial taxa, being able to produce many different biologically active secondary metabolites, including antibiotics, anticancer and immunosuppressant drugs, and degrading organic matter enzymes (Seipke et al., 2012). They have been reported to be intimately associated with the spores of a number of different AMF (Agnolucci et al., 2015). For instance, *Streptomyces* spp. showed promising PGP traits, being capable of solubilising phytates and phosphates, and producing indolacetic acid and siderophores (Battini et al., 2016). Moreover, wheat plants grown in soil contaminated by the pathogenic fungus *Rhizoctonia solani*, inoculated with *Streptomyces* sp. F5, showed lower root damage and higher grain yield, compared to untreated controls (Barnett et al., 2019). Species of *Rhodococcus* inoculated in wheat showed interesting PGP activities, increasing straw and grain weights, compared to non-inoculated control (Basheer et al., 2016), while some isolates from wheat rhizosphere enhanced plant sulphur availability by desulfonating carbon-bound sulfonate-sulphur (Schmalenberger et al., 2009). Interestingly, when inoculated in durum wheat, a strain of *Microbacterium* sp. promoted height and fresh weight (Yu et al., 2016), *Microbacterium foliorum* increased the stem diameter and the number of leaves (Valenzuela-Aragon et al., 2019) and *Microbacterium arborescens* increased plant height and grain uptake of N, P, Cu, Zn, Mn, and Fe, compared to controls (Kumar et al., 2017).

The relative abundance of some species belonging to Sphingomonadaceae (e.g., *Sphingomonas koreensis*) was the highest in Saragolla wheat roots inoculated with *F. mosseae*. The N-fixing functional trait of *Sphingomonas* spp. is of particular interest, suggesting their possible role in the promotion of plant growth. Actually, the inoculation of wheat seeds with a strain of *Sphingomonas* sp. increased root biomass accumulation and the concentration of nutrients (Xu et al., 2018).

*Chryseobacterium* was able to colonise Odisseo and Saragolla roots when the seeds were inoculated with AMF alone or in combination with LAB. Some species belonging to such genus showed interesting PGP traits: for example, *Chryseobacterium soldanellicola*, isolated from rhizosphere soil, was antagonist against *R. solani* (Yin et al., 2013), while a bioformulation based on poultry feather and *Chryseobacterium gleum*, used in salinity stressed wheat, produced higher roots and shoots length, fresh and dry weight, compared to non-inoculated plants (Bhise et al., 2017).

Arbuscular mycorrhizal fungi inoculation increased the abundance of Bacteroidetes in both wheat cultivars, as clearly revealed by PCR-DGGE sequencing, in particular *Ohtaekwangia koreensis* and *Niastella populi*, two species frequently retrieved from the soil (Zhang et al., 2010; Yoon et al., 2011). Moreover, inoculation of the AMF increased the abundance of the Proteobacteria *Pantoea*, a genus encompassing strains producing siderophores, thus increasing Fe bioavailability for wheat plants (Moreira et al., 2016). Interestingly, the species *Pantoea agglomerans*, retrieved here as wheat root endophyte, increased Zn bioavailability in wheat (Kamran et al., 2017), while a selected strain of *Pantoea alhagi* colonised wheat roots and enhanced wheat resistance to drought (Chen et al., 2017). AMF inoculation also increased the abundance of another bacterium belonging to Proteobacteria, *Brevundimonas* sp., which, previously isolated from wheat rhizosphere, showed PGP traits (Rana et al., 2012; Verma et al., 2015; Siddiqa et al., 2016).

## Conclusion

In conclusion, our work for the first time showed that AMF (*F. mosseae*), which are widely utilised as biofertilizers, and/or LAB (*L. plantarum*) represent significant ecological drivers in the establishment of the bacterial endophytic community in durum wheat roots. The increase of several bacterial species and genera encompassing PGP strains, promoted by wheat inoculation of LAB and, especially, AMF, represents a starting point for the exploitation of beneficial endophytes of wheat roots. Further studies are necessary to confirm the results shown in this paper and to better understand the dynamics of endophytes establishment in wheat roots, as modulated by the complex interactions among AMF, mycorrhizosphere bacteria and wheat roots.

## Data Availability Statement

The datasets generated for this study can be found in the Sequence Read Archive of NCBI, PRJNA543396.

## Author Contributions

MA coordinated plant and PCR-DGGE experimental activities and wrote the manuscript. MP performed the PCR-DGGE and statistical analyses on results from PCR-DGGE. CC performed the experiments with microorganisms inoculated in plants. NC extracted DNA and elaborated the results from 16S metagenetics. MGi conceived the study and wrote the manuscript. MD revised the manuscript. MGo conceived the study. FM coordinated the 16S metagenetics experimental activities and wrote the manuscript.

## Conflict of Interest

The authors declare that the research was conducted in the absence of any commercial or financial relationships that could be construed as a potential conflict of interest.

## Footnotes

1 http://blast.ncbi.nlm.nih.gov/Blast.cgi

2 http://www.megasoftware.net/
