# Supplementary material for: Beneficial Plant Microorganisms Affect the Endophytic Bacterial Communities of Durum Wheat Roots as Detected by Different Molecular Approaches
Source: Front Microbiol. 2019 Oct 31;10:2500. doi: 10.3389/fmicb.2019.02500 (PMC6834690; doi:10.3389/fmicb.2019.02500)
Supplement: Supplementary file 1 [file Data_Sheet_1.pdf]

Table S1. Estimated sample coverage and alpha diversity indexes of Bacteria (16S rRNA) found in roots of durum wheat (cultivars Odisseo, O, and Saragolla, S) inoculated with either *Lactobacillus plantarum* (O-B, S-B), or *Funneliformis mosseae* (O-M, S-M), or *L. plantarum* and *F. mossae* (O-BM, S-BM). Controls (O, S) refer to plants of durum wheat not inoculated.

Sample	Good's Estimated Sample Coverage (%)	Number of OTU	Chao1 richness	Abundance-based Coverage Estimate	Shannon diversity index
O	99.99	164	165.2	165.1	3.15
O-B	99.92	154	156.5	158.9	1.75
O-M	99.96	158	159.1	160.6	2.29
O-BM	99.94	116	117.8	120.5	1.37
S	99.98	135	135.4	135.8	3.49
S-B	99.99	156	156.8	157.4	3.42
S-M	99.97	163	164.2	165.8	3.53
S-BM	99.92	140	142.6	143.9	3.41

Figure S1. Venn's diagrams showing the number of shared and unique OTU within the roots of durum wheat of Odisseo (panel A) and Saragolla (panel B). O, non-inoculated Odisseo; O-B, Odisseo inoculated with *Lactobacillus plantarum*; O-M, Odisseo inoculated with *Funneliformis mosseae*; O-BM, Odisseo inoculated with *L. plantarum* and *F. mosseae*; S-B, Saragolla inoculated with *L. plantarum*; S-M, Saragolla inoculated with *F. mosseae*; S-BM, Saragolla inoculated with *L. plantarum* and *F. mosseae*.

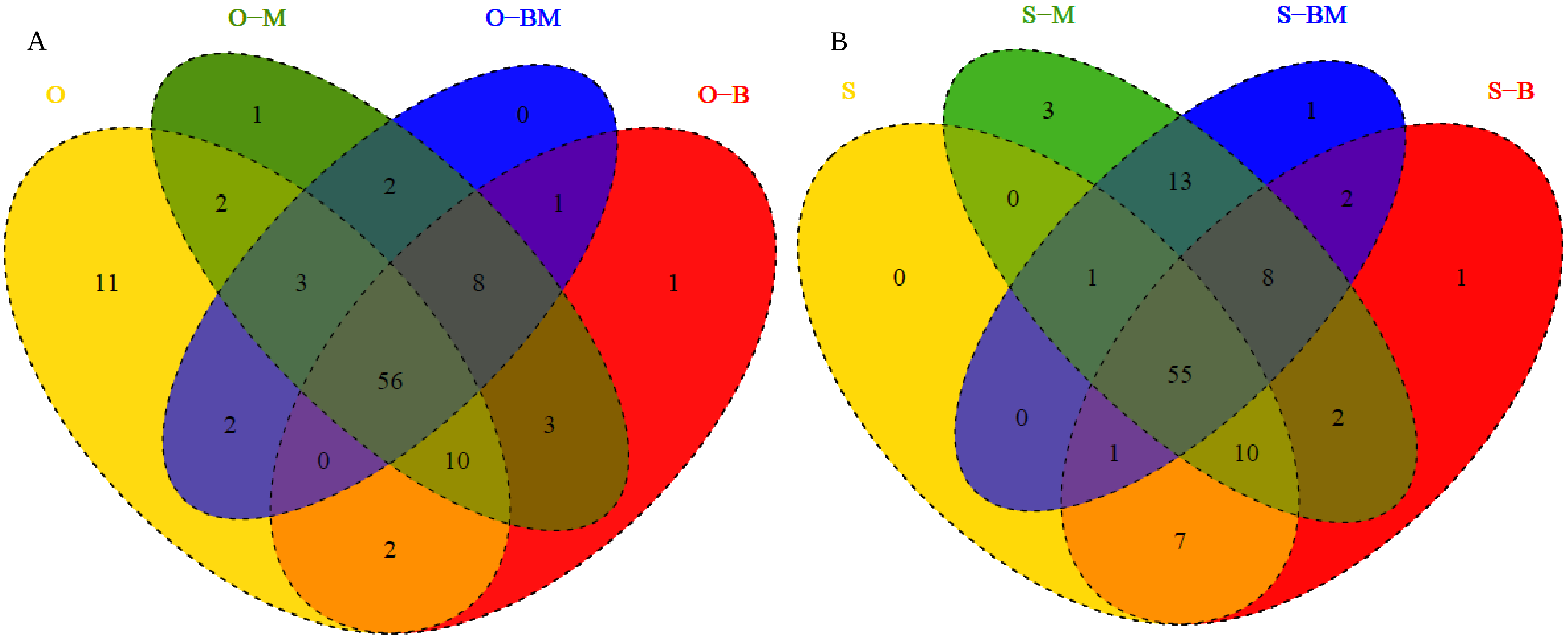

Table S2. Relative abundance (%)[a] of bacterial species/genera found through 16S metagenetic analysis of DNA extracted from roots of durum wheat (cultivars Odisseo, O, and Saragolla, S) inoculated with either *Lactobacillus plantarum* (O-B, S-B), or *Funneliformis mosseae* (O-M, S-M), or *L. plantarum* and *F. mossae* (O-BM, S-BM). Controls (O, S) refer to plant of durum wheat not inoculated.

Phylum/Family	Genus/Species	O	O-B	O-M	O-BM	S	S-B	S-M	S-BM
**Actinobacteria/Nocardiaceae**	*Rhodococcus erythropolis*	4.44	3.21	4.37	1.43	1.39	3.60	3.98	8.44
	Rhodococcus sp.	7.60	5.28	7.62	2.95	3.31	7.18	8.19	17.61
**Actinobacteria/Microbateriaceae**	*Curtobacterium* sp	0.13	0.03	0.02	0.04	0.35	0.07	0.74	0.06
	Microbacterium sp	0.34	0.92	0.97	0.75	0.15	0.60	1.14	1.12
	Microbacterium	0.16	0.06	0.07	0.01	0.12	0.10	0.01	0.05
**Actinobacteria/Micrococcaceae**	*Arthrobacter*	0.78	0.15	0.20	0.01	1.00	0.53	0.05	0.07
**Actinobacteria/Micromonosporaceae**	*Actinoplanes*	0.01	0.34	0.54	0.27	0.00	0.01	1.16	4.50
**Actinobacteria/Propionibacteriaceae**	*Propionibacterium acnes*	0.00	0.01	0.00	0.00	0.00	0.10	0.04	0.02
**Actinobacteria/Pseudonocardiaceae**	*Lentzea*	0.03	0.08	0.20	0.21	0.00	0.00	0.49	1.00
	Pseudonocardiaceae	0.00	0.01	0.03	0.06	0.00	0.00	0.19	0.29
**Actinobacteria/Streptomycetaceae**	*Streptomyces europaeiscabiei*	0.43	0.30	0.40	0.94	0.02	0.12	1.45	3.41
	Streptomyces galilaeus	0.00	0.02	0.01	0.00	0.00	0.00	0.17	0.12
	Streptomyces unk	0.03	0.07	0.28	0.03	0.00	0.03	0.42	0.41
**Bacteroidetes/Cyclobacteriaceae**	*Algoriphagus*	0.29	0.03	0.03	0.00	0.22	0.15	0.00	0.02
**Bacteroidetes/Cytophagaceae**	*Dyadobacter fermentans*	0.12	0.00	0.00	0.00	0.17	0.07	0.00	0.00
	Ohtaekwangia	0.41	0.07	0.10	0.05	0.19	0.29	0.17	1.16
	Cytophagales	0.42	0.03	0.00	0.00	0.22	0.35	0.06	0.04
**Bacteroidetes/Flavobacteriaceae**	*Chryseobacterium*	0.00	0.01	0.05	0.14	0.00	0.00	0.89	0.06
**Bacteroidetes/Chitinophagaceae**	*Chitinophaga*	0.48	0.21	0.03	0.00	2.17	1.04	1.54	0.10
	Niabella ginsengisoli	0.01	0.00	0.00	0.02	0.02	0.01	0.23	0.00
	Niastella unk	0.70	0.48	0.39	0.30	0.53	0.74	1.05	2.02
**Bacteroidetes/Sphingobacteriaceae**	*Pedobacter* sp	0.40	0.03	0.03	0.01	0.18	0.10	0.01	0.01

	Sphingobacterium sp	0.50	0.05	0.04	0.01	0.21	0.32	0.04	0.01
**Bacteroidetes**	*Bacteroidetes* unk	0.38	1.19	2.01	1.09	0.24	0.62	8.78	4.38
**Firmicutes/Bacillaceae**	*Bacillus* sp	0.17	0.00	0.01	0.00	0.15	0.14	0.01	0.00
**Proteobacteria/Caulobacteraceae**	*Asticcacaulis*	2.25	1.05	1.46	0.46	1.74	3.32	1.52	4.03
	Brevundimonas sp	0.96	0.46	0.58	0.43	1.62	0.81	6.43	1.77
	Caulobacter	0.10	0.12	0.14	0.03	0.02	0.10	0.19	0.10
	Phenylobacterium sp	0.29	0.05	0.05	0.03	0.19	0.48	0.11	0.13
**Proteobacteria/Bradyrhizobiaceae**	*Bosea* sp	0.14	0.09	0.09	0.06	0.11	0.12	0.41	0.17
	Bradyrhizobium sp	0.45	0.23	0.38	0.09	0.38	0.27	0.28	0.42
**Proteobacteria/Hyphomicrobiaceae**	*Devosia riboflavina*	2.03	0.48	0.69	0.51	6.66	2.23	2.97	0.97
	Devosia sp	3.61	1.91	3.24	0.88	9.62	6.17	3.11	3.03
	Devosia unk	0.50	0.03	0.24	0.01	0.33	1.04	0.09	0.11
	Hyphomicrobium sp	0.02	0.11	0.16	0.07	0.05	0.11	0.25	0.29
**Proteobacteria/Rhizobiaceae**	*Rhizobium* sp	2.01	1.58	3.65	1.14	10.09	4.73	2.48	9.49
	Rhizobium unk	3.61	0.56	1.07	0.56	6.64	5.10	1.72	1.50
	Shinella zoogloeoides	0.23	0.23	0.34	0.50	0.81	0.28	0.28	0.79
	Sinorhizobium meliloti	0.58	0.19	0.44	0.13	0.70	0.53	0.05	0.47
	Rhizobiaceae unk	1.41	0.56	0.97	0.34	4.34	2.61	1.43	0.93
**Proteobacteria**	*Rhizobiales* unk	2.48	1.09	1.74	1.07	7.61	2.71	2.24	4.21
**Proteobacteria/Rhodospirillaceae**	*Phaeospirillum*	0.15	0.04	0.08	0.02	0.46	0.11	0.14	0.00
**Proteobacteria/Sphingomonadaceae**	*Novosphingobium* sp	0.00	0.01	0.02	0.00	0.12	0.00	0.69	0.60
	Rhizorhapis suberifaciens	0.00	0.01	0.02	0.02	0.00	0.00	0.04	0.13
	Sphingobium cloacae	0.11	0.00	0.01	0.02	0.11	0.02	0.01	0.00
	Sphingobium yanoikuyae	0.08	0.03	0.03	0.00	0.31	0.20	0.00	0.00
	Sphingomonas koreensis	0.07	0.09	0.18	0.06	0.06	0.02	2.83	0.44

	Sphingomonas sp	0.14	0.01	0.00	0.00	0.34	0.13	0.01	0.03
	Sphingopyxis sp	0.27	0.06	0.04	0.02	0.26	0.43	0.42	0.10
**Proteobacteria/Alcaligenaceae**	*Achromobacter* sp	0.04	0.10	0.06	0.10	0.00	0.06	0.00	0.91
**Proteobacteria/Burkholderiaceae**	*Ralstonia* sp	1.32	0.91	0.34	0.00	1.60	1.16	0.09	0.03
**Proteobacteria/Comamonadaceae**	*Acidovorax* sp	0.03	0.04	0.07	0.05	0.00	0.36	0.21	0.11
	Hydrogenophaga intermedia	1.79	0.16	0.07	0.07	1.45	1.71	0.33	0.28
	Pseudorhodoferax	0.00	0.03	0.12	0.08	0.00	0.00	0.49	0.40
	Variovorax	1.42	0.10	0.04	0.00	1.11	0.42	0.01	0.04
**Proteobacteria/Oxalobacteraceae**	*Duganella* sp	1.16	0.20	0.18	0.06	7.87	4.61	0.09	0.10
	Herbaspirillum	0.11	0.00	0.00	0.00	0.02	0.07	0.03	0.02
	Massilia sp	1.93	0.15	0.04	0.00	1.65	0.97	0.00	0.03
	Massilia unk	0.29	0.03	0.06	0.09	0.39	0.01	0.01	0.09
**Proteobacteria/Rhodocyclaceae**	*Methyloversatilis* sp	0.11	0.00	0.00	0.00	0.27	0.22	0.00	0.00
**Proteobacteria/Cellvibrionaceae**	*Cellvibrio* sp	0.00	0.74	2.00	0.60	0.00	0.01	0.73	0.13
	Cellvibrio unk	0.01	0.16	0.18	0.16	0.00	1.39	0.52	0.18
**Proteobacteria/Enterobacteriaceae**	*Pantoea agglomerans*	3.24	0.17	0.14	0.64	10.24	1.66	15.56	0.16
**Proteobacteria/Pseudomonadaceae**	*Pseudomonas* sp	34.78	67.14	53.68	75.57	2.64	23.45	0.53	0.61
	Pseudomonas unk	1.00	0.25	0.27	1.61	0.16	1.86	4.99	0.14
**Proteobacteria/Xanthomonadaceae**	*Lysobacter yangpyeongensis*	1.01	0.23	0.14	0.03	0.59	0.51	0.09	0.06
	Pseudoxanthomonas sp	0.08	0.08	0.08	0.09	0.12	0.04	0.22	0.37
	Stenotrophomonas sp	0.13	0.33	0.23	1.52	0.09	0.27	1.96	1.64
	Xanthomonadaceae unk	0.86	0.25	0.10	0.08	1.34	0.64	0.07	0.05
**Tenericutes/Acoleplasmataceae**	*Sugarcane phytoplasma*	3.68	2.17	2.10	1.35	1.98	5.67	5.14	2.77

[a] Only OTUs with a relative abundance > 0.1% in at least one thesis are shown.
